# Machine learning prediction of hepatic steatosis using body composition parameters: A UK Biobank Study

**DOI:** 10.1038/s41514-023-00127-z

**Published:** 2024-01-09

**Authors:** Delbert Almerick T. Boncan, Yan Yu, Miaoru Zhang, Jie Lian, Varut Vardhanabhuti

**Affiliations:** 1 Snowhill Science Ltd, Units 801-803, Level 8, Core C, Hong Kong SAR, China; 2 School of Life Sciences, The Chinese University of Hong Kong, Shatin, New Territories, Hong Kong SAR, China; 3 Department of Diagnostic Radiology, Li Ka Shing Faculty of Medicine, The University of Hong Kong, Hong Kong SAR, China

**Keywords:** Obesity, Metabolic syndrome, Biomarkers

## Abstract

Non-alcoholic fatty liver disease (NAFLD) has emerged as the most prevalent chronic liver disease worldwide, yet detection has remained largely based on surrogate serum biomarkers, elastography or biopsy. In this study, we used a total of 2959 participants from the UK biobank cohort and established the association of dual-energy X-ray absorptiometry (DXA)-derived body composition parameters and leveraged machine learning models to predict NAFLD. Hepatic steatosis reference was based on MRI-PDFF which has been extensively validated previously. We found several significant associations with traditional measurements such as abdominal obesity, as defined by waist-to-hip ratio (OR = 2.50 (male), 3.35 (female)), android-gynoid ratio (OR = 3.35 (male), 6.39 (female)) and waist circumference (OR = 1.79 (male), 3.80 (female)) with hepatic steatosis. Similarly, A Body Shape Index (Quantile 4 OR = 1.89 (male), 5.81 (female)), and for fat mass index, both overweight (OR = 6.93 (male), 2.83 (female)) and obese (OR = 14.12 (male), 5.32 (female)) categories were likewise significantly associated with hepatic steatosis. DXA parameters were shown to be highly associated such as visceral adipose tissue mass (OR = 8.37 (male), 19.03 (female)), trunk fat mass (OR = 8.64 (male), 25.69 (female)) and android fat mass (OR = 7.93 (male), 21.77 (female)) with NAFLD. We trained machine learning classifiers with logistic regression and two histogram-based gradient boosting ensembles for the prediction of hepatic steatosis using traditional body composition indices and DXA parameters which achieved reasonable performance (AUC = 0.83–0.87). Based on SHapley Additive exPlanations (SHAP) analysis, DXA parameters that had the largest contribution to the classifiers were the features predicted with significant association with NAFLD. Overall, this study underscores the potential utility of DXA as a practical and potentially opportunistic method for the screening of hepatic steatosis.

## Introduction

Previously, the term non-alcoholic fatty liver disease (NAFLD) has been used to encompass a spectrum of liver pathology with macrovesicular steatosis in at least 5% of hepatocytes in individuals with low to no alcohol consumption. Non-alcoholic fatty liver (NAFL) or simple steatosis is the non-progressive subtype that does not usually have serious implications, although it is estimated that 25% of individuals with NAFLD develop non-alcoholic steatohepatitis (NASH)^1^—a progressive subtype that eventually advances to fibrosis, cirrhosis (ca. 25% of those with NASH)^2^, and hepatocellular carcinoma (HCC). Studies have shown that the presence and severity of NAFLD are associated with increased incidence and prevalence of cardiovascular disease (CVD) and chronic kidney disease (CKD)^3–13^. Notwithstanding the morbidity, mortality, and limited therapeutics of NAFLD-related cirrhosis and HCC, disease mortality is often seen as a result of type 2 diabetes mellitus (T2DM) and CVD complications^14,15^. While the aetiology remains to be fully understood, NAFLD is recognised as the hepatic manifestation of the metabolic syndrome^16^. Hence, the causal link of NAFLD to chronic morbidities (i.e., obesity, hypertension, T2DM, CVD, and CKD) is hypothesised—underscoring the concept of NAFLD as a multisystem disease with potential involvement in the pathology of extra-hepatic diseases^17^.

With NAFLD being closely associated with obesity and metabolic syndrome, its incidence and prevalence are increasing to epidemic proportions and becoming the most common cause of abnormal serum aminotransferase levels, chronic liver disease, and liver transplantation in the United States (US)^18–20^. Data in Asia also shows that NAFLD is as common and important as in the West, albeit it manifests at a lower body mass index (BMI) with many patients not displaying insulin resistance^21–23^. This ethnic variability including the differences in severity and rate of progression as a function of environmental risk exposures demonstrates that NAFLD is a complex disease trait^24^.

Lifestyle modification, as with other chronic diseases, is the cornerstone of NAFLD management regardless of the disease stage, so while end-stage liver disease has a poor prognosis, NAFLD is clinically manageable at its early onset. Classifying NAFLD into grades is imperative, especially in patients with advanced fibrosis who are at greater risk of developing complications of end-stage liver disease. Although invasive and costly, liver biopsy is still the gold standard in NASH diagnosis and NAFLD staging. While surrogate serum biomarkers exist for NASH, there have been no non-invasive tests that can reliably differentiate it from NAFL^18,25^. Ultrasonography, while lacking sensitivity, is used as the first-line screening of steatosis. Other imaging techniques such as controlled attenuated parameter (CAP) and computed tomography (CT) are promising, whilst magnetic resonance imaging—proton density fat fraction technique (MRI-PDFF) is considered by many as the gold standard. Considerations on sensitivity, efficiency, operator-dependent results, ease of operation, access, availability, and cost among others remain as limiting factors for these modalities, particularly limiting their potential utility in longitudinal and epidemiology-based studies^25^. The current understanding of NAFLD pathogenesis, its epidemiology and the available diagnostic strategies underscore the importance of thorough surveillance, early detection, and timely interventions (e.g., lifestyle modification) not only for epidemiological surveillance but also to address the risk of comorbidity in NAFLD patients.

DXA is an imaging technique that has been used commonly for assessing bone density. It also allows for body composition assessment particularly relating to muscle and fat deposition in the body. It is based on an X-ray imaging technique, with low radiation dose, and has been validated extensively for both bones and body composition analyses^26–28^. Besides the commonly used bone density measurements, body composition-related parameters that can be derived from DXA include visceral adipose tissue mass, total body fat percentage, fat-free mass, as well as muscle-related mass. In total, a DXA scan can give up to 48 different parameters pertaining to body composition. The major limitation of DXA, however, is the lack of representation of the body as a true 3D structure. Volumetric parameters are therefore estimates of the 2D projection measurements. Meanwhile, accuracy validations have shown that DXA-estimated mass with scale weight is within 1%^28–30^. Furthermore, DXA has been shown to correlate well with CT and MRI—cross-sectional imaging techniques which serve as gold standards in body composition assessment^31^. While this limitation exists, there has been a reported consensus in which DXA is considered a reference technique or at least a surrogate to CT/MRI for the assessment of body composition in clinical practice^32,33^.

Given the known relationships between NAFLD and body composition-related parameters such as visceral fat, we reasoned that using body composition-related parameters based on DXA imaging, a prediction model can be derived to predict people at risk of hepatic steatosis. To aid in this task, we first performed association analyses of various DXA-derived parameters and traditional body composition indices. The reference standard for hepatic steatosis for this study is taken as measurements on MRI using the PDFF techniques, which have been extensively validated previously to be comparable to histopathology^34–36^. We demonstrated that several DXA-derived parameters were significantly associated with hepatic steatosis. We then leverage the use of machine learning (ML) to identify the potential of hepatic steatosis and to classify them into grades based on DXA scan and body composition-related indices. Our hypothesis is that an accurate prediction model can be built to predict the risk of NAFLD based on DXA parameters.

## Results

### Cohort characteristics

**Fig. 1:**
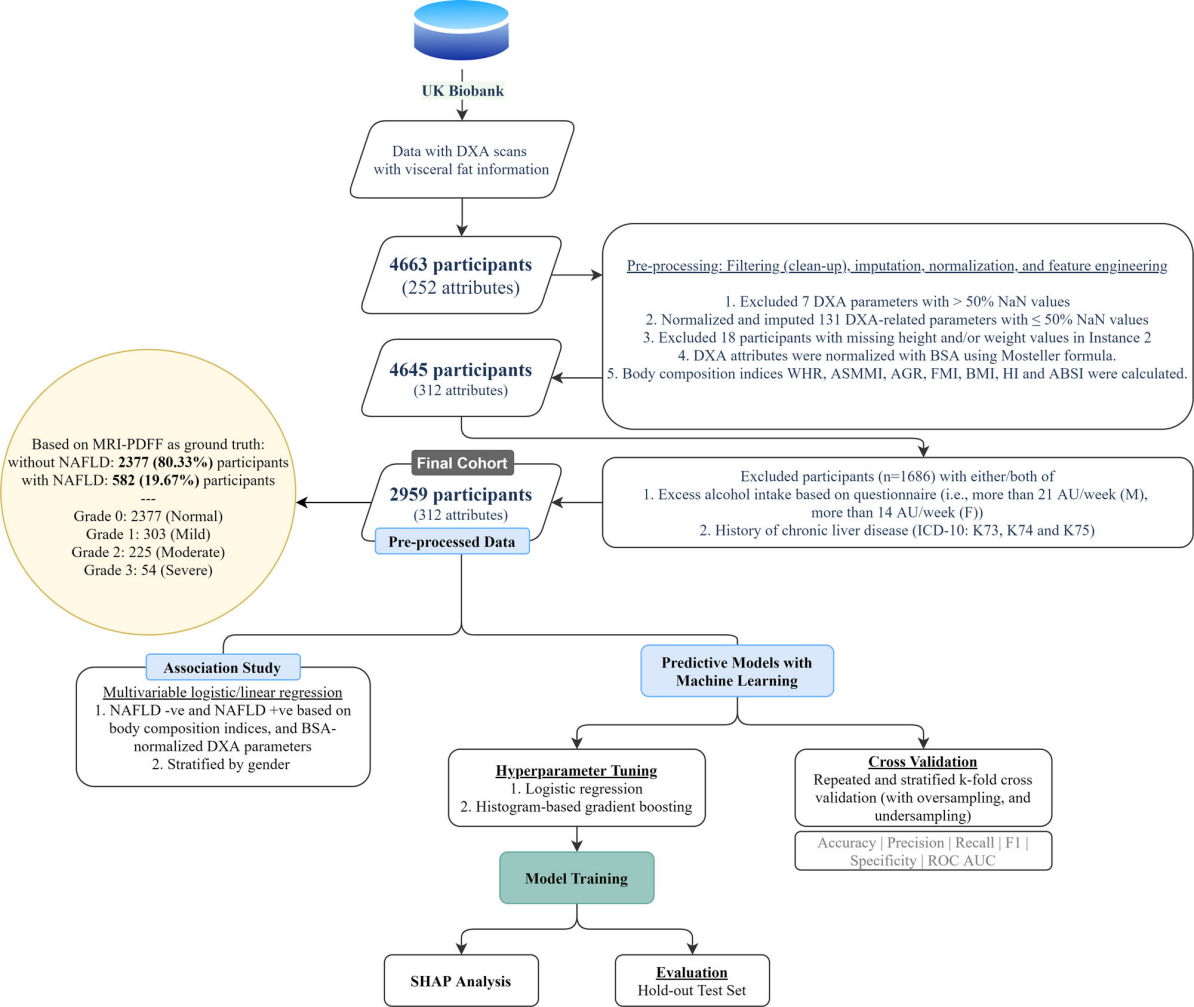
Overview of included data cohorts from the UK Biobank population and patient selection study workflow. DXA dual-energy X-ray absorptiometry, NaN null values, WHR waist-to-hip ratio, ASMMI appendicular skeletal muscle mass index, AGR android gynoid ratio, FMI fat mass index, BMI body mass index, HI hip index, ABSI A Body Shape Index, MRI-PDFF magnetic resonance imaging proton density fat fraction, NAFLD non-alcoholic fatty liver disease, ICD International Classification of Diseases, ROC receiver operating characteristic, AUC area under the curve, SHAP SHapley Additive exPlanations.

A total of 2959 participants remained after exclusion (see Table 1, Fig. 1). These were 1271 males and 1688 females. In this cohort, 582 participants (19.67%) were deemed as having NAFLD based on the liver MRI-PDFF^37^. In total 303 were classified as grade 1, 225 as grade 2, and 54 as grade 3, respectively. The characteristics of the cohorts are summarized in Table 1. When stratified by gender, there were significant differences between all the DXA-derived body composition indexes and BSA-normalized DXA parameters between the NAFLD +ve and NAFLD -ve groups (see Supplementary Tables 1 and 2).

**Table 1:** Descriptive statistics of body composition indices stratified by gender.

	Male (*n* = 1271)					Female (*n* = 1688)				
	NAFLD- (*n* = 960)		NAFLD+ (*n* = 311)			NAFLD- (*n* = 1417)		NAFLD+ (*n* = 271)		
	Mean	SD	Mean	SD	*P*	Mean	SD	Mean	SD	*P*
**Age**	62.57	7.76	62.08	7.79	<0.01	61.13	7.50	62.39	7.09	<0.01
**ABSI**	0.08	0.00	0.08	0.00	<0.01	0.07	0.00	0.08	0.00	<0.01
**AGR**	0.68	0.18	0.87	0.14	<0.01	0.43	0.13	0.62	0.13	<0.01
**ASMMI**	7.94	0.89	8.65	1.03	<0.01	6.42	0.81	7.07	0.93	<0.01
**BMI**	25.65	3.33	29.81	4.12	<0.01	25.39	4.25	30.63	4.87	<0.01
**FMI**	7.10	2.43	10.01	2.71	<0.01	9.55	3.21	13.48	3.53	<0.01
**HC**	99.95	6.61	105.70	7.63	<0.01	100.17	9.01	108.58	10.59	<0.01
**HI**	99.04	3.73	97.35	4.10	<0.01	105.02	4.06	104.49	4.28	<0.01
**WC**	90.60	8.91	100.86	10.30	<0.01	80.06	10.17	94.03	10.69	<0.01
**WHR**	0.91	0.05	0.95	0.06	<0.01	0.80	0.06	0.87	0.06	<0.01

### Association analysis

The multivariable logistic regression analysis of the body composition indices reveals several parameters to be significantly associated with hepatic steatosis (see Table 2). Of note, obesity defined as BMI over 25 yielded an odds ratio (OR) of 1.9 for males and 2.62 for females. Abdominal obesity, as defined by WHR (OR = 2.50 (male), 3.35 (female)), AGR (OR = 3.35 (male), 6.39 (female)) and WC (OR = 1.79 (male), 3.80 (female)) were all associated with hepatic steatosis. Similarly, when examining ABSI into different quartiles, the higher quartiles yielded the highest OR (Quantile 4 OR = 1.89 (male), 5.81 (female)), and for FMI, both the overweight (OR = 6.93 (male), 2.83 (female)) and the obese (OR = 14.12 (male), 5.32 (female)) categories were significantly associated with hepatic steatosis. When looking at DXA parameters, there were several parameters that were significantly associated with hepatic steatosis. A summary of the top 10 features is shown in Table 3 (with full results in Supplementary Table 3). Of note, we observed the biggest contribution from VAT mass (OR = 8.37 (male), 19.03 (female)), VAT volume (OR = 8.37 (male), 19.03 (female)), trunk fat mass (OR = 8.64 (male), 25.69 (female)), android fat mass (OR = 7.93 (male), 21.77 (female)) and total fat mass (OR = 3.60 (male), 3.90 (female)).

**Table 2:** NAFLD-associated body composition indices based on multivariable logistic regression analysis stratified by gender and adjusted by age, weight, and height.

	Male			Female		
Predictor (Quantiles, Min-Max for Male and Female)	Odds Ratio	95% Confidence Interval		Odds Ratio	95% Confidence Interval	
**Abdominal Obesity**, **WHR** **>** **0.9 (men), WHR** **>** **0.85 (women)**						
Normal	1			1		
Obese	2.50***	1.72	3.64	3.35***	2.49	4.51
**ABSI**						
Quantile 1 (0.064434-0.075964, 0.061029-0.070002)	1			1		
Quantile 2 (0.075993-0.078676, 0.070008-0.072982)	0.94	0.62	1.42	1.85*	1.08	3.16
Quantile 3 (0.078706-0.081124, 0.072985-0.076279)	1.40	0.92	2.13	3.54***	2.17	5.76
Quantile 4 (0.081130-0.096132, 0.076282-0.091292)	1.89**	1.24	2.89	5.81***	3.60	9.36
**ASMMI**, **kg/m**^**2**^						
Low	1					
High	1.20	0.58	2.52	1.01	0.61	1.66
**FMI**, **kg/m**^**2**^						
Normal (men: 3-6, women: 5-9)	1			1		
Overweight (male: > 6-9, women: > 9-13)	6.93***	3.18	15.13	2.83**	1.56	5.14
Obese (men > 9, women > 13)	14.12***	5.94	33.60	5.32***	2.52	11.22
**HC**, **cm**						
Quantile 1 (82-97, 77-94)	1			1		
Quantile 2 (98-101, 95-100)	1.00	0.59	1.69	1.19	0.62	2.29
Quantile 3 (102-105, 101-107)	0.74	0.41	1.32	0.69	0.33	1.45
Quantile 4 (106-150, 108-147)	1.14	0.63	2.09	0.61	0.27	1.36
**HI**						
Quantile 1 (85.73-96.14, 85.45-102.14)	1			1		
Quantile 2 (96.16-98.75, 102.16-104.99)	0.88	0.60	1.29	0.70	0.47	1.03
Quantile 3 (98.75-100.95, 104.99-107.69)	0.76	0.51	1.14	0.56**	0.38	0.84
Quantile 4 (100.97-134.92, 107.70-121.83)	0.66	0.43	1.01	0.46***	0.30	0.69
**Overall Obesity**, **BMI** $\boldsymbol{\ge}$ **25**						
Normal	1			1		
Obese	1.90*	1.04	3.49	2.62**	1.45	4.76
**AGR**						
AGR $\le$ 1 (men), AGR $\le$ 0.8	1					
AGR > 1 (men), AGR > 0.8 (women)	3.35***	1.96	5.72	6.39***	2.56	5.65
**WC,** $\boldsymbol{\ge}$**102** **cm/90** **cm (non-Asian/Asian men),** $\boldsymbol{\ge}$ **88** **cm/80** **cm (non-Asian/Asian women)**						
Low	1			1		
High	1.79**	1.21	2.66	3.80***	2.56	5.65

Significance: *P $$\le$$ 0.05, **P $$\le$$ 0.01, ***P $$\le$$ 0.001.

**Table 3:** Top 5 positively NAFLD-associated DXA parameters with multivariable linear regression analysis stratified by gender and adjusted by age, weight, and height.

	Male (*n* = 1271)			Female (*n* = 1688)		
Predictor	Odds Ratio	95% Confidence Interval		Odds Ratio	95% Confidence Interval	
Android fat mass	7.93***	3.66	17.18	21.77***	11.42	41.48
Total fat mass	3.60**	1.46	8.84	3.90**	1.54	9.90
Trunk fat mass	8.64***	3.75	19.94	25.69***	12.80	51.58
VAT (visceral adipose tissue) mass	8.36***	4.59	15.23	19.03***	12.74	28.42
VAT (visceral adipose tissue) volume	8.37***	4.59	15.23	19.03***	12.75	28.42

Significance: *P $$\le$$ 0.05, **P $$\le$$ 0.01, ***P $$\le$$ 0.001

### Machine learning models and prediction

**Fig. 2:**
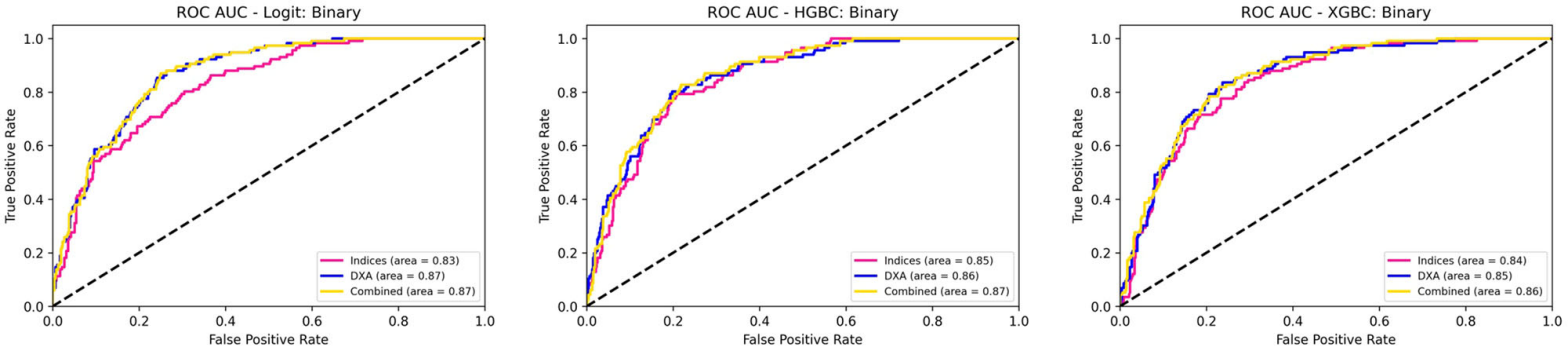
ROC AUC curves for the three different machine learning classifications. ROC receiver operating characteristic, AUC area under the curve, LR logistic regression, HGBC HistGradient Boosting Classifier, XGBC Extreme Gradient Boosting, DXA dual-energy X-ray absorptiometry.

We set out to compare 3 machine learning classifiers. In binary classification, all three achieved reasonable performance with ROC AUC = 0.83-0.87 (Fig. 2). Supplementary tables 4-7 show the full results with separate evaluations using cross-fold validation and hold-out test validation sets. In this main section, we discuss the results of the hold-out test set with the graphical comparison of the 3 models on the hold-out test set shown in Fig. 2. We shall discuss the results of HGBC binary classification in more detail. Using the body composition indices, HGBC achieved an AUC of 0.8519, sensitivity of 0.7601, and specificity of 0.7500. Using DXA-parameters, HGBC achieved an AUC of 0.8617, sensitivity of 0.7736, and specificity of 0.7605. Using combined parameters, HGBC achieved an AUC of 0.8656, sensitivity of 0.7686, and specificity of 0.7542. Using a combination of traditional body composition indices and DXA parameters did not improve performance. Multiclass classification models performed reasonably well in NAFLD grading (Supplementary Fig. 2). For example, using HGBC on DXA parameters, a weighted average ROC AUC (wROCUC) of 0.8377 was achieved, with class 0 (AUC = 0.86), class 1 (AUC = 0.72), class 2 (AUC = 0.79) and class 3 (AUC = 0.70), respectively. In addition, gender-specific binary classification models had similar or better performance for females (Supplementary Figs. 3 and 4). For example, with HGBC, body composition indices (AUC = 0.86), DXA-parameters (AUC = 0.88), and combined (AUC = 0.89).

**Fig. 3:**
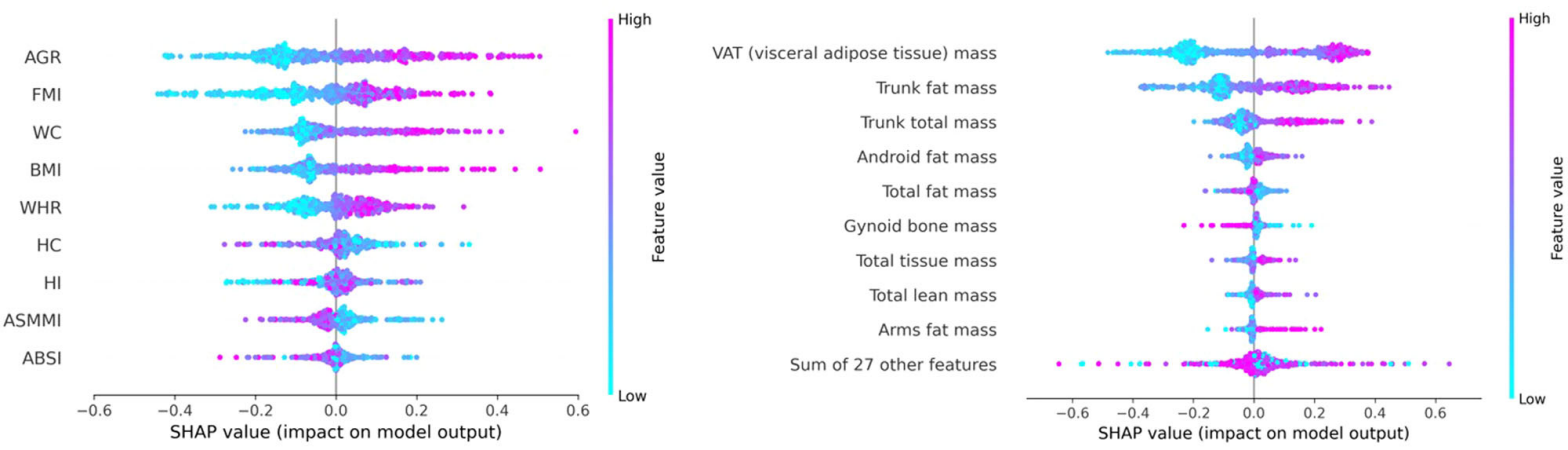
SHAP feature importance on body composition indices (left) and BSA-normalised DXA parameters (right). SHAP SHapley Additive exPlanations, BSA body surface area, AGR android gynoid ratio, FMI fat mass index, BMI body mass index, WC waist circumference, WHR waist-to-hip ratio, HC hip circumference, HI hip index, ASMMI appendicular skeletal muscle mass index, ABSI A Body Shape Index.

We then proceeded to examine the contribution of each of the features using SHAP analysis. All SHAP analyses for the 3 classifiers are demonstrated in Supplementary Figs. 5-10. The SHAP features for HGBC and XGBC were almost identical. For the main result section, we shall focus on HGBC. As expected, the top contributions from the machine learning models were from the features that were highly associated with hepatic steatosis based on the odds ratio (Fig. 3). For example, the top 3 contributions from body composition analyses were from AGR, FMI, and WC. Whereas for the BSA-normalised DXA parameters, the top 3 contributions were from VAT mass, trunk fat mass, and trunk total mass.

**Fig. 4:**
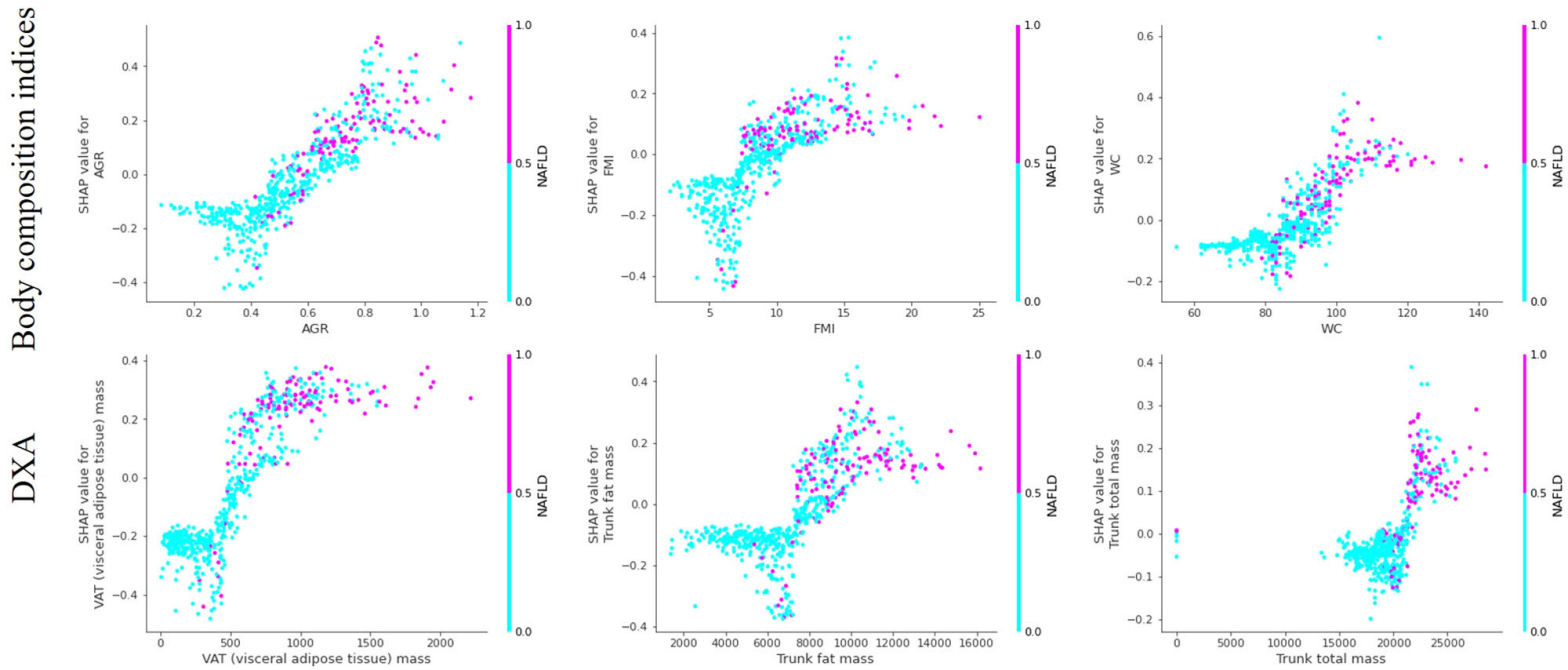
SHAP dependence plots of the top 3 predictors of HGBC models trained on body composition indices and DXA parameter. HGBC HistGradient Boosting Classifier, AGR android gynoid ratio, SHAP SHapley Additive exPlanations, BSA DXA, dual-energy X-ray absorptiometry, NAFLD non-alcoholic fatty liver disease.

The SHAP dependency plots are shown in Fig. 4 for the top 3 contributions. There are clear positive correlations between increasing SHAP values and increasing risks of disease with more distinct separations between the low and high-risk groups.

## Discussion

We have shown that DXA-derived parameters were highly associated with hepatic steatosis as measured on MRI-PDFF. Within the traditional body composition indices, FMI (which utilises fat mass information from DXA scan) has the strongest association compared to other traditional metrics. Previously, it has been shown that traditional metrics such as WC were shown to be predictive for hepatic steatosis and fibrosis^38^ but we have shown in our study that FMI was more predictive. Other studies have also highlighted the importance of DXA parameters such as AG ratio and VAT mass^39^ but we believe our study is the first to compare all the parameters with traditional parameters such as WC. A recent study also demonstrated that FMI can be used with high accuracy to identify hepatic steatosis as determined by ultrasonography with a high degree of accuracy^40^. With regards to DXA, we have shown that many DXA parameters (normalised to BSA) were highly associated with hepatic steatosis, not limiting to fat-related parameters which would be expected, but also other parameters such as those relating to lean mass. For instance, the total lean mass has an odds ratio of less than 1 for both genders indicating a negative association with NAFLD. Lee et al. (2021) observed that participants in their study had less skeletal muscle mass over several years of follow-up, and their findings suggest that maintaining muscle mass is important in NAFLD management^41^. Meanwhile, Cho et al. (2022) have shown that skeletal muscle mass to visceral fat area ratio could serve as a complementary index to conventional adiposity indices in detecting NAFLD among lean yet overweight men and women^42^. This underscores the potential and practical application of non-conventional indices or measurements to NAFLD diagnosis—not only limited to adiposity indices. There are several studies that have examined the role of muscle mass (particularly fat infiltration of muscle), and we also wanted to examine some of the other parameters relating to muscle that can be derived from DXA scans. Whilst some associations were seen between some of the lean mass parameters on DXA, by far the strongest associations were observed in parameters pertaining to fat, with an extremely strong association with VAT mass and volume, trunk fat mass, android fat mass, and total mass, far higher than those seen using traditional parameters. With several parameters on DXA being associated with hepatic steatosis, we set out to build a machine learning model that can be used to predict hepatic steatosis, and we showed that a reasonably accurate model can be built using these parameters.

In this study, we utilised logistic regression and 2 boosting classifiers. As expected, the performance and feature importance of classifiers varied slightly. On one hand, LR performed marginally better than HGBC with DXA parameters in the gender-unstratified dataset (Fig. 2). On the other hand, gender-stratified-trained models show that histogram-based boosting classifiers outperformed LR with body composition indices but not with DXA parameters (Supplementary Figs. 3 and 4). Theoretically, LR is less robust in high-dimensional datasets where it tends to overfit as opposed to boosting classifiers. While LR could be trained with DXA parameters, the assumption of linearity between dependent and independent variables is a major limitation. Furthermore, the existence of multicollinearity between DXA parameters is expected which makes boosting ensemble classifier a more suitable algorithm that can estimate all types of relationships between dependent and independent variables. In cases where LR performed better, we hypothesize that it is because of the default regularisation in LR. With regularisation, the performance, and accuracy of the LR model are improved by reducing overfitting and underfitting. Furthermore, it also addresses the issue of multicollinearity in LR. In general, DXA parameters outperformed traditional body composition indices in any ML algorithm. Meanwhile, combining body composition indices and DXA parameters did not result in a significant improvement in performance. We hypothesised that this could be due to the more encompassing nature of DXA parameters than traditional body composition indices. While a minimum number of DXA parameters based on association and feature importance could be inferred, the infinitesimal yet cumulative importance of other parameters cannot be discounted.

Early detection of NAFLD is important in order that timely intervention can be prescribed to patients (e.g., lifestyle and diet modification) by healthcare practitioners. In this study, DXA-based ML models demonstrate a potential alternative means to perform early diagnosis of NAFLD, although it is important to take note that the results presented are preliminary and are subject to follow-up validations. Moreover, accessibility to DXA scanning needs to be borne in mind. Nevertheless, the performance of the models based on ROC AUC and sensitivity makes them a promising surrogate compared to conventional imaging techniques. Ultrasonography, for instance, has a sensitivity greater than 90% if the fat content is higher than 30%. Similarly, CT achieves 82% sensitivity on moderate to severe degrees of steatosis^43,44^. Meanwhile, MRI has a sensitivity of 80-95.8% making it the gold standard in the detection of liver steatosis^35,45,46^. While these imaging techniques can all be considered suitable for early detection of NAFLD, concerns on detection limit, radiation exposure (in case of CT), access, and ease of operation among others have resulted in divided preferences on their adoption in the clinical practice to quantify liver steatosis. To this end, liver biopsy has remained the gold standard in confirming NASH. However, due to its invasiveness, the frequency of patient/participant hesitating and subsequent refusal to undergo the procedure may exceed 50% in some centres—ostensibly precluding its potential utility as a practical option in early NAFLD screening or detection^47^.

There are some limitations worth noting. First, we recognise that the recently proposed metabolic-associated fatty liver disease (MAFLD) is now recommended for usage with the aim to cover the more heterogeneous nature of the disease, and not excluding the impact of alcohol on the disease^48^. For the purpose of this study, we set out to examine and isolate the metabolic associated factors and hence have excluded patients with excess alcoholic intake. Second, the data used for this study was from the UK Biobank cohort, and whilst this is useful for the predominantly Western population, applicability to other regions and ethnicity may need to be further examined. Third, we did not have an independent validation set to test the generalisability of our model beyond the UK Biobank cohort. We are currently in the process of recruiting participants to pursue this objective, so we can test the generalisability of our findings.

As NAFLD cases rise to epidemic proportions, new tools that can potentially be used as opportunistic screening may be helpful particularly as early detection is important. In this study, we not only showed the association of traditional body composition indices to hepatic steatosis but also the strong association of DXA parameters to hepatic steatosis. As expected, visceral adipose tissue mass, trunk fat mass, and adipose tissue mass showed a strong positive association with hepatic steatosis, while total lean mass also demonstrated a negative association. The ML models trained on two types of predictors are practical applications of how body composition indices and DXA can potentially be leveraged to opportunistically screen for NAFLD although more prospective studies with validation across different populations as well as cost-effective analysis need to be performed before this can be adopted more widely.

## Methods

The data used were from the UK Biobank which received ethical approval from the North West Multicentre Research Ethics Committee (REC reference: 11/NW/03820). All participants gave written informed consent before enrolment in the study. This research has been conducted using the UK Biobank Resource under Application Number 78730. Additionally, this study was approved by the authors’ own local ethics board (UW-20814) at the University of Hong Kong.

### Study population

The UK Biobank cohort consists of over half a million participants from the general population in the United Kingdom (UK). Participants were aged between 40 and 70 years at enrolment and were recruited between 2006 and 2010, with follow-up data. In 2014, the imaging assessments were performed on these cohorts with the aim of collecting 100,000 participants with imaging of the brain, cardiac and abdominal magnetic resonance imaging, DXA, and carotid ultrasound. At the time of writing, the UK Biobank imaging project has collected imaging scans from over 60,000 participants (https://www.ukbiobank.ac.uk/explore-your-participation/contribute-further/imaging-study). For this study, we focused on the imaging data, particularly those with abdominal MRI and DXA imaging and retrieved all other relevant associated information. Only participants with MRI-PDFF^37,49^ (UK Biobank Category 126) and DXA-derived parameters including visceral fat were included. The UK Biobank provides an imaging modality (https://biobank.ctsu.ox.ac.uk/crystal/crystal/docs/DXA_explan_doc.pdf) for DXA as a reference.

### Data pre-processing

Data processing, statistics, machine learning classification and visualization were performed with custom-made Python scripts based on Statsmodels and Scikit-Learn unless stated otherwise^50,51^. Electronic health records were retrieved from participants in the UK Biobank limiting the search to those with “10 P Liver PDFF (proton density fat fraction) | Instance 2” and “VAT (visceral adipose tissue) mass”. The downloaded dataset includes DXA-related attributes with additional attributes on gender, age, alcohol consumption, and comorbidities. In summary, a total of 4663 participants were retrieved from the UK Biobank with matching records. DXA-related attributes with more than 50% missing values were excluded (*n* = 7), while participants with less than 50% missing DXA attributes were imputed with multiple imputation by chained equations (MICE)^52,53^. Likewise, participants with missing height and/or weight attributes in Instance 2 were excluded (*n* = 18). This resulted in 4645 remaining participants. DXA attributes were normalized with body surface area (BSA) using Mosteller formula^54^. The choice of Mosteller formula to calculate BSA was based on its accuracy in various clinical use-case scenarios and applicability among normal, overweight, and obese adults^55–60^. Body composition indices including waist-to-hip ratio (WHR), appendicular skeletal muscle mass index (ASMMI), android gynoid ratio (AGR), fat mass index (FMI), BMI, hip index (HI), and a body shape index (ABSI) were calculated. National Health And Nutrition Examination Survey (NHANES) population average values for {height} = 166 cm, {weight} = 73 kg were used for calculating ABSI^38,61,62^.

### Reference standard, predictor variables and inclusion criteria

While liver biopsy remains the gold standard in NAFLD diagnosis and grading, its inherent invasiveness limits it from routine use. The proton density fat fraction in MRI (MRI-PDFF) has been demonstrated to correlate well with total lipid accumulation in the liver and thus making it a suitable surrogate and reference standard for liver biopsy^34–36^. In this study, UK Biobank participants were categorized into NAFLD grades (0-1 – absence-presence or 0-3 – normal, mild, moderate, severe as class labels) based on the MRI-PDFF values following Szczepaniak et al.’s NAFLD grading scheme (cut-off values)^63^. In brief, the grading scheme 0, 1, 2 and 3 corresponds to 0-$\le$5.56%, 5.56%-$\le$10%, 10%-$\le$20%, and >20% fat content (steatosis), respectively^34,64^. Participants with excess alcohol intake or known chronic liver disease were excluded, defined as either consuming more than 21 (Male) or 14 alcohol units (Female) per week (*n* = 1654), with chronic liver diseases (International Classification of Diseases, Tenth Revision ICD-10: K73, K74 and K75) (*n* = 18), or both (*n* = 14)^65–68^. Considering both alcohol intake habits and the presence/absence of chronic liver disease, the total number of participants in the final cohort is 2959.

### Statistical analysis

Two sets of predictor variables were adopted for the analysis: (1) 9 body composition indices and (2) 36 (mass- and volume-based) BSA-normalized DXA parameters. We set out to determine the association between the different variables with hepatic steatosis. Independent sample t-tests with unequal variances were performed to determine whether the two groups (NAFLD- and NAFLD + ) in this study exhibit significant differences in various predictor variables. Multivariable adjusted (with age, weight, and height) analysis with logistic regression with respective odds ratios was performed to rank categories or quantiles (body composition indices) with respect to case-control (“normal”) or to the first quantile of the sample^69^. Similarly, odds ratios for DXA parameters were calculated from the standardized (beta, β) coefficients of linear regression analysis.

### Machine learning model training and evaluation

We then set out to develop ML prediction models for the prediction of hepatic steatosis. Three machine learning classifiers were compared. Logistic regression (LR), two histogram-based gradient boosting ensembles: HistGradientBoostingClassifier (HGBC, Scikit-Learn), and Extreme Gradient Boosting (XGBoost) classifier (XGBC) ensemble algorithms were employed to train binary and multiclass classifiers taking inputs of body composition indices, BSA-normalised DXA values or combined variables (body composition indices and BSA-normalized DXA)^70,71^. Data was randomly partitioned into 80:20 train-test sets with stratification such that the proportions of NAFLD +ve and NAFLD -ve were consistent in both sets. Owing to imbalanced datasets, boosting techniques of the minority class were used^72^. The minority classes were oversampled with the synthetic minority oversampling technique—support vector machine (SMOTE-SVM) (*k*, *m* = 10, 5). Meanwhile, the majority class was re-sampled and under-sampled in the process with the synthetic minority oversampling technique—edited nearest neighbour (SMOTE-ENN) and RandomUnderSampler, respectively^72^. Hyperparameters were optimised for specificity based on *k*-1 validation sets while simultaneously testing for performance with repeated (*n* = 3) and stratified *k*-folds (*k* = 10) cross-validation. For LR, the solver and tolerance parameters were optimized for specificity (and in all other algorithms with L2 regularisation parameters). For HBGC, optimisation parameters included a maximum number of leaves for each tree, the maximum depth of each tree, and a minimum number of samples per leaf. For XGBC optimisation parameters included learning rate, number of estimators, maximum tree depth, lambda regularisation, and subsample ratio of the training instances. Models were built using optimized hyperparameters with SMOTE-oversampled minority class/es on the hold-out train sets. Supplementary Table 8 lists the optimised hyperparameters for various models we trained for this study, while Supplementary Tables 4 and 5 show the performance metrics of ML algorithms trained with different types of predictors on gender-(un)stratified sets. Model performance was evaluated on a separate hold-out test dataset for (area under the curve of the receiver operating characteristic) various performance metrics. Finally, feature importance was identified and ranked based on SHAP values^73^.

### Reporting summary

Further information on research design is available in the Nature Research Reporting Summary linked to this article.

### Supplementary information

Reporting Summary

Supplementary information

## Data Availability

The data used for this study is from the UK Biobank, and data access needs to be requested and approved directly from the individual’s institution. The data regarding training, validation, and test datasets will not be available from the corresponding author for sharing owing to the restriction imposed by the UK Biobank which precludes sharing of their data with other investigators.

## Code availability

Codes used for this study are available upon reasonable request from the authors.
