# Supplementary material for: Machine learning prediction of hepatic steatosis using body composition parameters: A UK Biobank Study
Source: NPJ Aging. 2024 Jan 9;10(1):4. doi: 10.1038/s41514-023-00127-z (PMC10776620; doi:10.1038/s41514-023-00127-z)
Supplement: Supplementary file 1 — Reporting Summary [file 41514_2023_127_MOESM1_ESM.pdf]

Corresponding author(s): V Vardhanabhuti

Last updated by author(s): Oct 9, 2023

Reporting Summary

Nature Portfolio wishes to improve the reproducibility of the work that we publish. This form provides structure for consistency and transparency in reporting. For further information on Nature Portfolio policies, see our Editorial Policies and the Editorial Policy Checklist.

Statistics

For all statistical analyses, confirm that the following items are present in the figure legend, table legend, main text, or Methods section.

n/a	Confirmed	
☐	☒	The exact sample size (*n*) for each experimental group/condition, given as a discrete number and unit of measurement
☐	☒	A statement on whether measurements were taken from distinct samples or whether the same sample was measured repeatedly
☐	☒	The statistical test(s) used AND whether they are one- or two-sided<br>*Only common tests should be described solely by name; describe more complex techniques in the Methods section.*
☐	☒	A description of all covariates tested
☐	☒	A description of any assumptions or corrections, such as tests of normality and adjustment for multiple comparisons
☐	☒	A full description of the statistical parameters including central tendency (e.g. means) or other basic estimates (e.g. regression coefficient) AND variation (e.g. standard deviation) or associated estimates of uncertainty (e.g. confidence intervals)
☐	☒	For null hypothesis testing, the test statistic (e.g. F, t, r) with confidence intervals, effect sizes, degrees of freedom and P value noted<br>*Give P values as exact values whenever suitable.*
☒	☐	For Bayesian analysis, information on the choice of priors and Markov chain Monte Carlo settings
☒	☐	For hierarchical and complex designs, identification of the appropriate level for tests and full reporting of outcomes
☒	☐	Estimates of effect sizes (e.g. Cohen's *d*, Pearson's *r*), indicating how they were calculated

Our web collection on statistics for biologists contains articles on many of the points above.

Software and code

Policy information about availability of computer code

Data collection	No specific software was used.
Data analysis	Python scripts based on Statsmodels and Scikit-Learn

For manuscripts utilizing custom algorithms or software that are central to the research but not yet described in published literature, software must be made available to editors and reviewers. We strongly encourage code deposition in a community repository (e.g. GitHub). See the Nature Portfolio guidelines for submitting code & software for further information.

Data

Policy information about availability of data

All manuscripts must include a data availability statement. This statement should provide the following information, where applicable:

- Accession codes, unique identifiers, or web links for publicly available datasets
- A description of any restrictions on data availability
- For clinical datasets or third party data, please ensure that the statement adheres to our policy

Provide your data availability statement here.

Research involving human participants, their data, or biological material

Policy information about studies with human participants or human data. See also policy information about sex, gender (identity/presentation), and sexual orientation and race, ethnicity and racism.

Reporting on sex and gender	We report gender specific breakdown on the data where appropriate for the study.
Reporting on race, ethnicity, or other socially relevant groupings	The study ethnicity breakdown was detailed as appropriate. Socially relevant groupings were not considered specifically as was not considered relevant for the study. Specific patient characteristics namely history of chronic liver disease, and level of alcohol intake were considered in exclusion of participants.
Population characteristics	See above.
Recruitment	The recruitment for the UK biobank study was outlined and has been documented extensively previously.
Ethics oversight	Both the original UK Biobank study site and authors' own institution approved of the study.

Note that full information on the approval of the study protocol must also be provided in the manuscript.

Field-specific reporting

Please select the one below that is the best fit for your research. If you are not sure, read the appropriate sections before making your selection.

☒ Life sciences ☐ Behavioural & social sciences ☐ Ecological, evolutionary & environmental sciences

For a reference copy of the document with all sections, see nature.com/documents/nr-reporting-summary-flat.pdf

Life sciences study design

All studies must disclose on these points even when the disclosure is negative.

Sample size	Sample size was arrived at based on availability of the data. The final number of 2959 participants is considered sufficient based on prior similar studies.
Data exclusions	1. missing values<br>2. history of chronic liver disease<br>3. excess alcohol intake
Replication	The study is not directly replicable as these require long data collection time. The methods employ in this study is replicable based on experimental methodology outlined in the manuscript.
Randomization	Random stratified splitting was performed for machine learning prediction model.
Blinding	Blinding was not relevant as this was not an interventional study.

Reporting for specific materials, systems and methods

We require information from authors about some types of materials, experimental systems and methods used in many studies. Here, indicate whether each material, system or method listed is relevant to your study. If you are not sure if a list item applies to your research, read the appropriate section before selecting a response.

Materials & experimental systems

n/a	Involved in the study	
☒	☐	Antibodies
☒	☐	Eukaryotic cell lines
☒	☐	Palaeontology and archaeology
☒	☐	Animals and other organisms
☐	☒	Clinical data
☒	☐	Dual use research of concern
☒	☐	Plants

Methods

n/a	Involved in the study	
☒	☐	ChIP-seq
☒	☐	Flow cytometry
☒	☐	MRI-based neuroimaging

Clinical data

Policy information about clinical studies

All manuscripts should comply with the ICMJE guidelines for publication of clinical research and a completed CONSORT checklist must be included with all submissions.

Clinical trial registration	*Provide the trial registration number from ClinicalTrials.gov or an equivalent agency.*
Study protocol	North West Multicentre Research Ethics Committee (REC reference: 11/NW/03820)
Data collection	UK Biobank DXA (https://biobank.ctsu.ox.ac.uk/crystal/crystal/docs/DXA_explan_doc.pdf)
Outcomes	The original UK Biobank study is done as a longitudinal study with no specific primary or secondary outcome.<br>The primary outcome of this study was the accuracy of prediction of hepatic steatosis based on DXA parameters.
