# Supplementary material for: Machine learning prediction of hepatic steatosis using body composition parameters: A UK Biobank Study
Source: NPJ Aging. 2024 Jan 9;10(1):4. doi: 10.1038/s41514-023-00127-z (PMC10776620; doi:10.1038/s41514-023-00127-z)
Supplement: Supplementary file 2 — Supplementary information [file 41514_2023_127_MOESM2_ESM.pdf]

SUPPLEMENTARY

TABLES

Supplementary table 1. Independent sample t-test with equal variances of body composition indices (predictors) between NAFLD- and NAFLD+ gender-stratified groups.

	Male (n = 1271)			Female (n = 1688)		
**Predictor**	**NAFLD- σ^2**	**NAFLD+ σ^2**	***P***	**NAFLD- σ^2**	**NAFLD+ σ^2**	***P***
ABSI	0.000015	0.000016	3.65E-02	0.00002	0.000021	<0.001
AGR	0.03	0.02	<0.001	0.02	0.02	<0.001
ASMMI	0.79	1.06	<0.001	0.66	0.87	<0.001
BMI	11.06	16.95	<0.001	18.04	23.62	<0.001
FMI	5.88	7.33	<0.001	10.29	12.40	<0.001
HC	43.68	58.10	<0.001	81.11	111.76	<0.001
HI	13.88	16.76	<0.001	16.50	18.27	5.25E-02
WC	79.28	105.67	<0.001	103.33	113.77	<0.001
WHR	0.0028	0.0032	<0.001	0.0035	0.0036	<0.001

Supplementary table 2. Independent sample t-test with equal variances of BSA-normalized DXA parameters (predictors) between NAFLD- and NALFD+ gender-stratified groups.

	Male (n = 1271)			Female (n = 1688)		
**Predictor**	**NAFLD- σ^2**	**NAFLD+ σ^2**	***P***	**NAFLD- σ^2**	**NAFLD+ σ^2**	***P***
Android bone mass	32.72	31.29	3.54E-02	23.54	16.81	1.36E-01
Android fat mass	211483.78	181918.26	<0.001	216303.9	181245.35	<0.001
Android lean mass	33253.7	28564.13	3.24E-01	24133.63	31652.84	5.91E-02
Android total mass	214568.16	349364.37	<0.001	280601.17	497361.73	<0.001
Arm fat mass (left)	19418	28764.28	<0.001	41725.69	61350.07	<0.001
Arm fat mass (right)	19599.36	26623.97	<0.001	43416.82	57440.86	<0.001
Arm lean mass (left)	32347.45	29310.89	3.17E-02	16324.17	18543.22	1.16E-03
Arm lean mass (right)	34122.93	26247.26	2.22E-01	16684.28	14727.28	1.10E-01
Arm total mass (left)	632580.73	915716.97	7.46E-01	558353	759128.07	<0.001
Arm total mass (right)	697337.37	985278.83	9.95E-01	598542.12	780624.51	<0.001
Arms fat mass	77257.94	105536.53	<0.001	169269.87	231006.55	<0.001
Arms lean mass	121219.11	104506.73	7.22E-02	59617.82	70307.01	1.51E-02
Arms total mass	252208.27	458714.24	<0.001	420471.54	850776.35	<0.001
Gynoid bone mass	459.08	354.27	1.27E-03	277.86	258.17	4.84E-03
Gynoid fat mass	187439.81	218791.68	<0.001	355704.99	472034.84	<0.001
Gynoid lean mass	117519.26	110260.68	8.48E-01	71709.84	89642.76	<0.001
Gynoid total mass	352150.41	680941.46	<0.001	759478.47	1504364.05	4.48E-03
Leg fat mass (left)	153360.88	197378.39	<0.001	405690.36	523745.96	<0.001
Leg fat mass (right)	158872.54	192516.32	<0.001	412899.01	528727.41	<0.001
Leg lean mass (left)	167383.2	157349.88	1.26E-01	111178.07	102768.59	3.24E-02
Leg lean mass (right)	168078.5	151797.27	8.66E-02	112034.45	102536.27	1.43E-01
Leg total mass (left)	4936297.67	6843802.88	5.52E-01	5805399.88	6519460.48	5.86E-01
Leg total mass (right)	5095508.41	7027269.14	5.44E-01	5939941.69	6688618.96	5.42E-01
Legs fat mass	622258.22	756024.39	<0.001	1614117.21	2104877.95	<0.001
Legs lean mass	639226.67	591239.27	9.15E-02	424967.42	481135.16	3.97E-02
Legs total mass	1798508.71	3160026.64	<0.001	3935996.96	6916433.61	3.11E-01
Total fat mass	9529009.46	8864528.48	<0.001	13045731.78	11831176.5	<0.001
Total fat-free mass	3532631.28	2642470.6	2.11E-03	2525956.66	3174415.31	<0.001
Total lean mass	3322249.79	2491705.37	7.06E-03	2313436.43	2925138.48	<0.001
Total tissue mass	6832258.39	9684168.02	<0.001	9619722.18	15820502.51	<0.001
Total mass	13316613.36	27123461.06	<0.001	25544131.18	52667583.34	<0.001
Trunk fat mass	4992287	4137322.67	<0.001	5502127.85	4317659.26	<0.001
Trunk lean mass	1120193.44	732775.58	<0.001	875473.02	953366.35	<0.001
Trunk total mass	4948508.39	8878010.08	<0.001	7587307.3	15177828.6	<0.001
VAT (visceral adipose tissue) mass	132227.29	131729.26	<0.001	55952	68563.01	<0.001
VAT (visceral adipose tissue) volume	148569.16	148011.4	<0.001	62868.2	77042.56	<0.001

Supplementary table 3. Linear regression analysis of DXA parameters (Z-score standardized) and MRI-PDFF as outcome variable stratified based on gender and adjusted by age, weight and height.

	Male (n = 1271)			Female (n = 1688)		
**Predictor**	**Odds Ratio**	**95% Confidence Interval**		**Odds Ratio**	**95% Confidence Interval**	
Android bone mass	0.95	0.65	1.37	0.98	0.73	1.32
Android fat mass	7.93***	3.66	17.18	21.77***	11.42	41.48
Android lean mass	0.83	0.50	1.39	1.05	0.71	1.55
Android total mass	3.80**	1.67	8.64	2.77***	1.68	4.57
Arm fat mass (left)	1.51	0.81	2.81	2.24**	1.32	3.78
Arm fat mass (right)	1.49	0.80	2.77	2.07**	1.22	3.53
Arm lean mass (left)	0.67	0.39	1.14	1.11	0.75	1.63
Arm lean mass (right)	0.72	0.43	1.21	1.01	0.68	1.48
Arm total mass (left)	1.01	0.71	1.43	1.28	0.97	1.70
Arm total mass (right)	1.01	0.71	1.44	1.26	0.95	1.66
Arms fat mass	1.72	0.92	3.22	2.01*	1.16	3.48
Arms lean mass	0.72	0.42	1.22	1.02	0.68	1.51
Arms total mass	0.68	0.37	1.25	1.03	0.68	1.56
Gynoid bone mass	0.75	0.48	1.15	0.92	0.65	1.32
Gynoid fat mass	0.47*	0.23	0.97	0.16***	0.09	0.28
Gynoid lean mass	0.75	0.41	1.38	0.60*	0.38	0.95
Gynoid total mass	0.43*	0.21	0.86	0.36***	0.24	0.54
Leg fat mass (left)	0.37**	0.20	0.67	0.16***	0.10	0.26
Leg fat mass (right)	0.39**	0.22	0.71	0.15***	0.10	0.24
Leg lean mass (left)	0.39**	0.20	0.74	0.30***	0.18	0.49
Leg lean mass (right)	0.42**	0.22	0.79	0.32***	0.20	0.53
Leg total mass (left)	0.9	0.63	1.28	0.92	0.69	1.21
Leg total mass (right)	0.91	0.64	1.29	0.92	0.70	1.22
Legs fat mass	0.38**	0.21	0.68	0.15***	0.10	0.25
Legs lean mass	0.38**	0.20	0.74	0.30***	0.18	0.50
Legs total mass	0.25***	0.13	0.48	0.25***	0.17	0.38
Total fat mass	3.60**	1.46	8.84	3.90**	1.54	9.90
Total fat-free mass	0.37**	0.19	0.73	0.47**	0.29	0.75
Total lean mass	0.39**	0.20	0.76	0.48**	0.30	0.76
Total tissue mass	1.12	0.00	1.29E+03	0.69	0.11	4.22
Total mass	0.62	0.27	1.43	0.69	0.44	1.10
Trunk fat mass	8.64***	3.75	19.94	25.69***	12.80	51.58
Trunk lean mass	0.59	0.34	1.01	0.72	0.49	1.04
Trunk total mass	2.08	0.90	4.81	1.66*	1.05	2.63
VAT (visceral adipose tissue) mass	8.36***	4.59	15.23	19.03***	12.74	28.42
VAT (visceral adipose tissue) volume	8.37***	4.59	15.23	19.03***	12.75	28.42

Significance: $*P \leq 0.05$, $**P \leq 0.01$, $***P \leq 0.001$

Supplementary table 4. Repeated and stratified k-fold cross validation on various evaluation score metrics for binary classification with optimized hyperparameters based on specificity. Combined – body composition indices and DXA parameters.

LR

	Indices		DXA		Combined	
	Mean	SD	Mean	SD	Mean	SD
**accuracy**	0.7263	0.0294	0.7883	0.0245	0.7876	0.0237
**F1**	0.7227	0.0252	0.7702	0.0243	0.7707	0.0235
**precision**	0.8168	0.0171	0.8510	0.0139	0.8504	0.0136
**recall**	0.6921	0.0287	0.7458	0.0282	0.7464	0.0272
**ROC AUC**	0.7263	0.0294	0.7883	0.0245	0.7876	0.0237
**specificity**	0.6700	0.0362	0.7182	0.0377	0.7196	0.0366

HGBC

	Indices		DXA		Combined	
	Mean	SD	Mean	SD	Mean	SD
**accuracy**	0.7469	0.0359	0.7880	0.0236	0.7836	0.0282
**F1**	0.7641	0.0252	0.7863	0.0262	0.7846	0.0253
**precision**	0.8250	0.0205	0.8488	0.0127	0.8461	0.0157
**recall**	0.7410	0.0289	0.7652	0.0309	0.7634	0.0295
**ROC AUC**	0.7469	0.0359	0.7880	0.0236	0.7836	0.0282
**specificity**	0.7372	0.0329	0.7505	0.0407	0.7503	0.0370

XGBC

	Indices		DXA		Combined	
	Mean	SD	Mean	SD	Mean	SD
**accuracy**	0.74524	0.03571	0.78689	0.02323	0.78432	0.02844
**F1**	0.76590	0.02379	0.78445	0.02177	0.78383	0.02573
**precision**	0.82384	0.02042	0.84815	0.01298	0.84673	0.01606
**recall**	0.74341	0.02723	0.76298	0.02566	0.76242	0.03008
**ROC AUC**	0.74524	0.03571	0.78689	0.02323	0.78432	0.02844
**specificity**	0.74223	0.03181	0.74753	0.03460	0.74822	0.03842

Supplementary table 5. Repeated and stratified k-fold cross validation on various evaluation score metrics for binary classification with optimized hyperparameters based on specificity for male and female participants/subjects.

Male

LR

	Indices		DXA		Combined	
	Mean	SD	Mean	SD	Mean	SD
**accuracy**	0.7211	0.0437	0.7485	0.0413	0.7487	0.0428
**F1**	0.7105	0.0464	0.7235	0.0411	0.7238	0.0422
**precision**	0.7884	0.0294	0.8093	0.0286	0.8095	0.0297
**recall**	0.6903	0.0498	0.7037	0.0439	0.7041	0.0452
**ROC AUC**	0.7211	0.0437	0.7485	0.0413	0.7487	0.0428
**specificity**	0.6606	0.0682	0.6606	0.0587	0.6611	0.0602

HGBC

	Indices		DXA		Combined	
	Mean	SD	Mean	SD	Mean	SD
**accuracy**	0.7364	0.0449	0.7697	0.0481	0.7645	0.0489
**F1**	0.7277	0.0432	0.7541	0.0389	0.7507	0.0408
**precision**	0.7976	0.0306	0.8197	0.0335	0.8161	0.0337
**recall**	0.7086	0.0472	0.7369	0.0423	0.7333	0.0444
**ROC AUC**	0.7364	0.0449	0.7697	0.0481	0.7645	0.0489
**specificity**	0.6819	0.0609	0.7053	0.0471	0.7032	0.0500

XGBC

	Indices		DXA		Combined	
	Mean	SD	Mean	SD	Mean	SD
**accuracy**	0.7402	0.0467	0.7697	0.0501	0.7645	0.0436
**F1**	0.7353	0.0445	0.7555	0.0415	0.7528	0.0368
**precision**	0.7993	0.0312	0.8196	0.0342	0.8163	0.0305
**recall**	0.7171	0.0486	0.7385	0.0452	0.7359	0.0403
**ROC AUC**	0.7402	0.0467	0.7697	0.0501	0.7645	0.0436
**specificity**	0.6949	0.0616	0.7084	0.0504	0.7084	0.0529

Female

LR

	Indices		DXA		Combined	
	Mean	SD	Mean	SD	Mean	SD
**accuracy**	0.7401	0.0455	0.7986	0.0490	0.7968	0.0487
**F1**	0.7557	0.0270	0.7904	0.0302	0.7897	0.0288
**precision**	0.8447	0.0219	0.8719	0.0230	0.8711	0.0228
**recall**	0.7210	0.0319	0.7612	0.0357	0.7605	0.0340
**ROC AUC**	0.7401	0.0455	0.7986	0.0490	0.7968	0.0487
**specificity**	0.7120	0.0321	0.7435	0.0353	0.7432	0.0333

HGBC

	Indices		DXA		Combined	
	Mean	SD	Mean	SD	Mean	SD
**accuracy**	0.7685	0.0567	0.7987	0.0480	0.8021	0.0439
**F1**	0.7924	0.0322	0.8061	0.0242	0.8100	0.0224
**precision**	0.8566	0.0263	0.8708	0.0227	0.8722	0.0205
**recall**	0.7659	0.0379	0.7812	0.0284	0.7859	0.0260
**ROC AUC**	0.7685	0.0567	0.7987	0.0480	0.8021	0.0439
**specificity**	0.7646	0.0364	0.7729	0.0294	0.7782	0.0263

XGBC

	Indices		DXA		Combined	
	Mean	SD	Mean	SD	Mean	SD
**accuracy**	0.7680	0.0495	0.7917	0.0495	0.7929	0.0487
**F1**	0.7918	0.0264	0.8057	0.0242	0.8080	0.0210
**precision**	0.8564	0.0228	0.8674	0.0227	0.8679	0.0227
**recall**	0.7652	0.0313	0.7812	0.0282	0.7842	0.0242
**ROC AUC**	0.7680	0.0495	0.7917	0.0495	0.7929	0.0487
**specificity**	0.7638	0.0305	0.7761	0.0270	0.7799	0.0230

Supplementary table 6. Evaluation metrics of ML models on hold-out test sets

**Binary Classification**	**Logit**			**HGBC**			**XGBC**		
**Predictor / Metric**	**Indices**	**DXA**	**Combined**	**Indices**	**DXA**	**Combined**	**Indices**	**DXA**	**Combined**
Accuracy	0.7392	0.7905	0.7970	0.7759	0.7940	0.7909	0.7599	0.7908	0.7888
Precision	0.8251	0.8536	0.8582	0.8413	0.8515	0.8499	0.8326	0.8496	0.8489
Recall/Sensitivity	0.6959	0.7365	0.7365	0.7601	0.7736	0.7686	0.7449	0.7736	0.7652
Specificity	0.6681	0.7017	0.6975	0.7500	0.7605	0.7542	0.7353	0.7626	0.7500
F1	0.7268	0.7629	0.7631	0.7818	0.7939	0.7896	0.7685	0.7937	0.7867
*ROC AUC	0.8277	0.8679	0.8684	0.8519	0.8617	0.8656	0.8391	0.8544	0.8574

**Multiclass Classification**	**Logit**			**HGBC**			**XGBC**		
**Predictor / Metric**	**Indices**	**DXA**	**Combined**	**Indices**	**DXA**	**Combined**	**Indices**	**DXA**	**Combined**
Accuracy	-	-	-	0.3845	0.3709	0.3521	0.3790	0.4049	0.3633
Precision	-	-	-	0.7920	0.7992	0.7984	0.7918	0.8017	0.7973
Recall/Sensitivity	-	-	-	0.6402	0.6571	0.6588	0.6368	0.6740	0.6554
Specificity	-	-	-	0.7095	0.7347	0.7474	0.7053	0.7516	0.7368
F1	-	-	-	0.6962	0.7112	0.7137	0.6942	0.7233	0.7098
*ROC AUC	-	-	-	0.8242	0.8377	0.8392	0.8293	0.8313	0.8366

*ROC AUC were calculated as weighted ROC AUC (wROCAUC).
*wROCAUC ≈ Class 0, Class 1for binary classification

Supplementary table 7. Evaluation metrics of ML models on hold-out test sets in binary classification for male and female.

Male	Logit			HGBC			XGBC		
Predictor / Metric	Indices	DXA	Combined	Indices	DXA	Combined	Indices	DXA	Combined
Accuracy	0.7006	0.7855	0.7881	0.7607	0.7826	0.7775	0.7553	0.7927	0.7763
Precision	0.7756	0.8340	0.8353	0.8121	0.8304	0.8278	0.8078	0.8342	0.8214
Recall/Sensitivity	0.6627	0.7333	0.7373	0.7373	0.7373	0.7294	0.7373	0.7608	0.7608
Specificity	0.6269	0.6839	0.6891	0.7150	0.6943	0.6839	0.7202	0.7306	0.7461
F1	0.6867	0.7521	0.7557	0.7546	0.7555	0.7484	0.7543	0.7766	0.7756
ROC AUC	0.7883	0.8727	0.8731	0.8075	0.8472	0.8361	0.8131	0.8263	0.8280

Female	Logit			HGBC			XGBC		
Predictor / Metric	Indices	DXA	Combined	Indices	DXA	Combined	Indices	DXA	Combined
Accuracy	0.7538	0.8155	0.8027	0.8000	0.7938	0.7960	0.7965	0.8106	0.8000
Precision	0.8518	0.8792	0.8733	0.8711	0.8685	0.8693	0.8696	0.8761	0.8711
Recall/Sensitivity	0.7249	0.7781	0.7692	0.7899	0.8047	0.7959	0.7840	0.8077	0.7899
Specificity	0.7113	0.7606	0.7535	0.7852	0.8099	0.7958	0.7782	0.8063	0.7852
F1	0.7598	0.8048	0.7972	0.8133	0.8243	0.8176	0.8085	0.8277	0.8133
ROC AUC	0.8200	0.8848	0.8853	0.8599	0.8772	0.8853	0.8509	0.8779	0.8814

Supplementary table 8. Hyperparameters optimised for specificity.

Algorithm	Predictors	Classification	Gender	Hyperparameters
LR	Indices	binary	M	class_weight: balanced, solver: lbfgs, tol: 0.001, max_iter: 5000, C: 1e-5
LR	DXA	binary	M	class_weight: balanced, solver: lbfgs, tol: 0.001, max_iter: 5000, C: 1e-5
LR	Combined	binary	M	class_weight: balanced, solver: liblinear, tol: 0.001, max_iter: 5000, C: 1e-5
HGBC	Indices	binary	M	max_depth: 15, max_leaf_nodes: 31, min_samples_leaf: 15
HGBC	DXA	binary	M	max_depth: 10, max_leaf_nodes: 31, min_samples_leaf: 15
HGBC	Combined	binary	M	max_depth: 15, max_leaf_nodes: 37, min_samples_leaf: 18
XGBC	Indices	binary	M	learning_rate: 0.2, max_depth: 15, n_estimators: 75, reg_lambda: 1, subsample: 1
XGBC	DXA	binary	M	learning_rate: 0.5, max_depth: 15, n_estimators: 75, reg_lambda: 3, subsample: 1
XGBC	Combined	binary	M	learning_rate: 0.5, max_depth: 10, n_estimators: 175, reg_lambda: 3, subsample: 0.6
LR	Indices	binary	F	class_weight: balanced, solver: lbfgs, tol: 0.001, max_iter: 5000, C: 1e-5
LR	DXA	binary	F	class_weight: balanced, solver: liblinear, tol: 0.001, max_iter: 5000, C: 1e-5
LR	Combined	binary	F	class_weight: balanced, solver: lbfgs, tol: 0.001, max_iter: 5000, C: 1e-5
HGBC	Indices	binary	F	max_depth: 15, max_leaf_nodes: 33, min_samples_leaf: 24
HGBC	DXA	binary	F	max_depth: 15, max_leaf_nodes: 35, min_samples_leaf: 21
HGBC	Combined	binary	F	max_depth: 25, max_leaf_nodes: 47, min_samples_leaf: 18
XGBC	Indices	binary	F	learning_rate: 0.3, max_depth: 15, n_estimators: 75, reg_lambda: 3, subsample: 0.8
XGBC	DXA	binary	F	learning_rate: 0.5, max_depth: 5, n_estimators: 175, reg_lambda: 1, subsample: 0.8
XGBC	Combined	binary	F	learning_rate: 0.5, max_depth: 15, n_estimators: 200, reg_lambda: 2, subsample: 0.8
LR	Indices	binary	M/F	class_weight: balanced, solver: lbfgs, tol: 0.001, max_iter: 5000, C: 1e-5
LR	DXA	binary	M/F	class_weight: balanced, solver: liblinear, tol: 0.001, max_iter: 5000, C: 1e-5
LR	COmbined	binary	M/F	class_weight: balanced, solver: lbfgs, tol: 0.001, max_iter: 5000, C: 1e-5
HGBC	Indices	binary	M/F	max_depth: 15, max_leaf_nodes: 45, min_samples_leaf: 15
HGBC	DXA	binary	M/F	max_depth: 20, max_leaf_nodes: 47, min_samples_leaf: 18
HGBC	Combined	binary	M/F	max_depth: 25, max_leaf_nodes: 47, min_samples_leaf: 15
HGBC	Indices	multiclass	M/F	max_depth: 20, max_leaf_nodes: 49, min_samples_leaf: 30
HGBC	DXA	multiclass	M/F	max_depth: 40, max_leaf_nodes: 49, min_samples_leaf: 39
HGBC	Combined	multiclass	M/F	max_depth: 30, max_leaf_nodes: 49, min_samples_leaf: 36
XGBC	Indices	binary	M/F	learning_rate: 0.4, max_depth: 10, n_estimators: 200, reg_lambda: 1, subsample: 0.2
XGBC	DXA	binary	M/F	learning_rate: 0.4, max_depth: 15, n_estimators: 100, reg_lambda: 1, subsample: 0.8
XGBC	Combined	binary	M/F	learning_rate: 0.5, max_depth: 10, n_estimators: 150, reg_lambda: 1, subsample: 1
XGBC	Indices	multiclass	M/F	learning_rate: 0.2, max_depth: 20, n_estimators: 175, reg_lambda: 3, subsample: 0.8
XGBC	DXA	multiclass	M/F	learning_rate: 0.5, max_depth: 15, n_estimators: 200, reg_lambda: 3, subsample: 0.6
XGBC	Combined	multiclass	M/F	learning_rate: 0.4, max_depth: 25, n_estimators: 175, reg_lambda: 2, subsample: 0.8

FIGURES

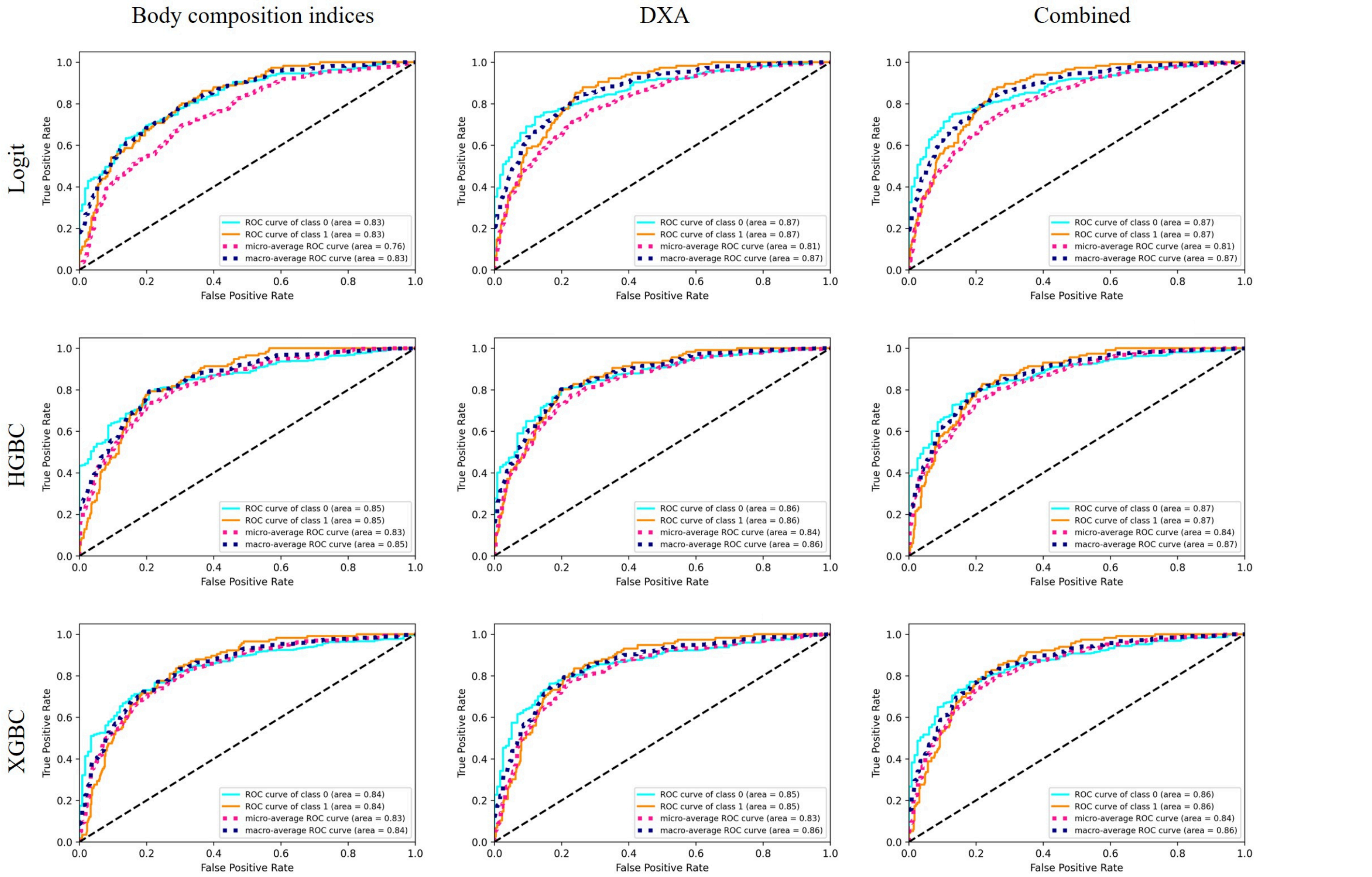

Supplementary Figure 1. ROC AUC of LR, HGBC and XGBC on binary classification using body composition indices, DXA parameters and combined body composition indices and DXA parameters (combined). Evaluation is based on hold-out test sets.

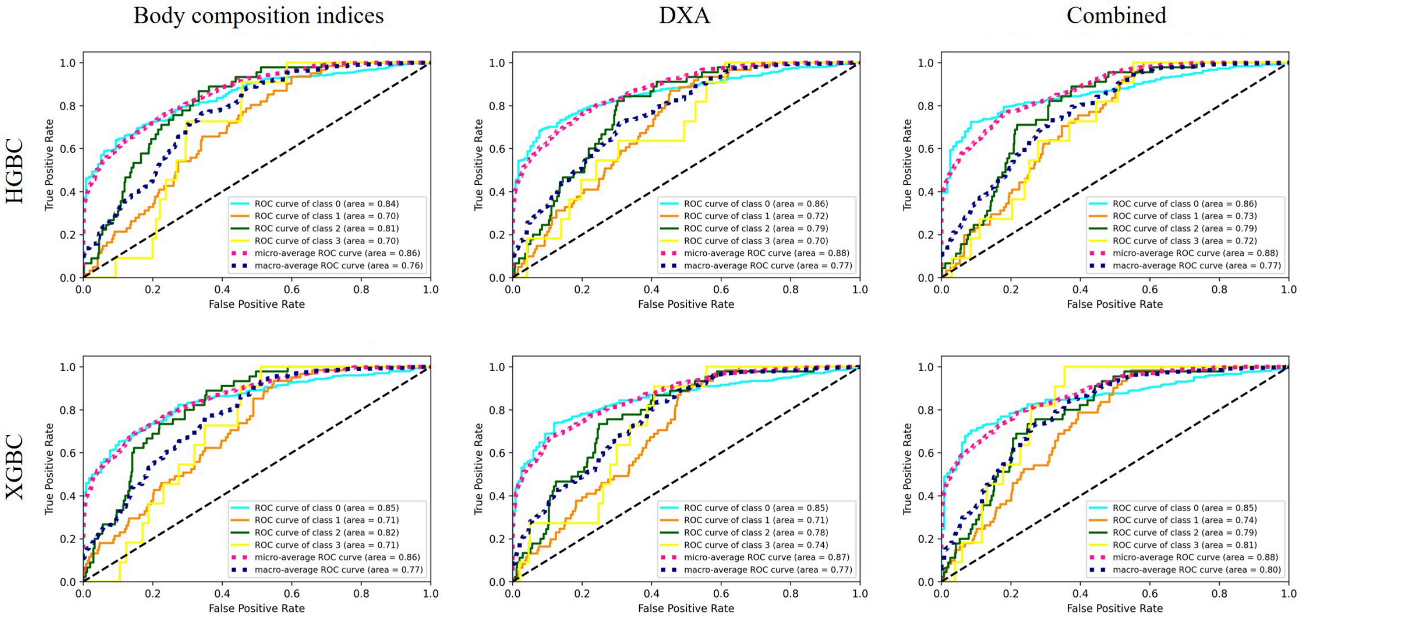

Supplementary Figure 2. ROC AUC of HGBC and XGBC on multiclass classification (NAFLD grades) evaluated based on hold-out test sets. NAFLD grades 0, 1, 2, 3 refer to normal, mild, moderate, and severe grades, respectively.

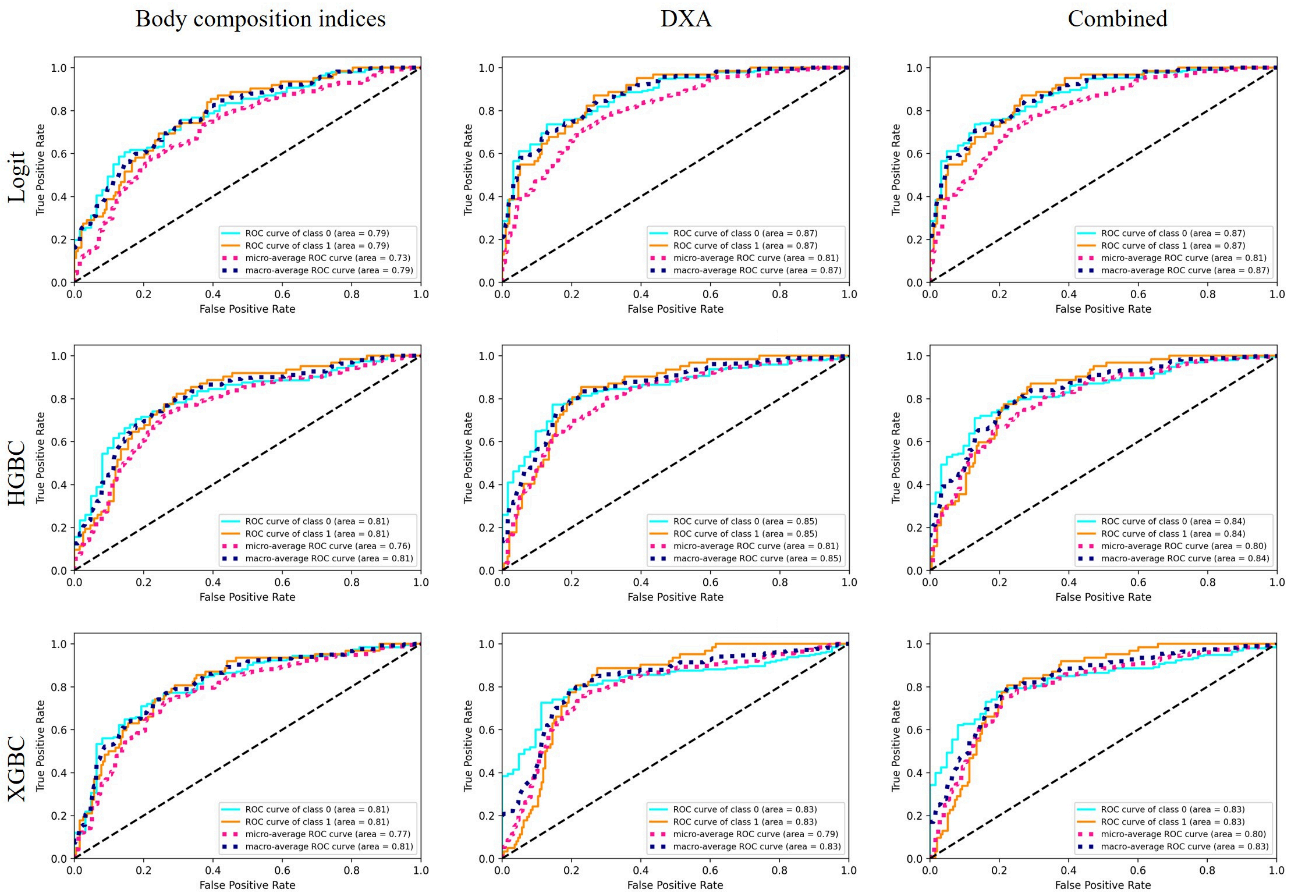

Supplementary Figure 3. ROC AUC of gender-stratified models (male) on binary classification evaluated on hold-out test sets.

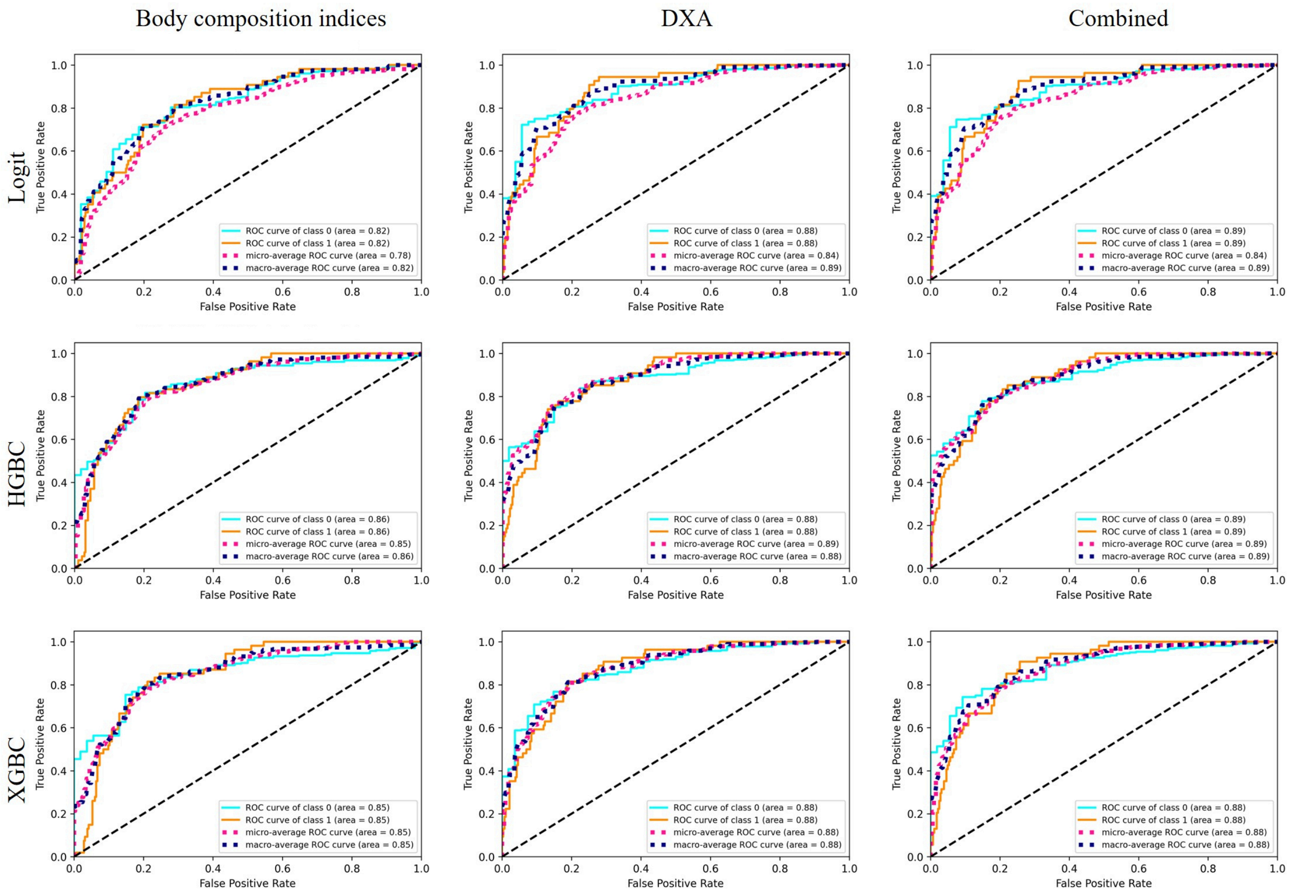

Supplementary Figure 4. ROC AUC of gender-stratified models (female) on binary classification evaluated on hold-out test sets.

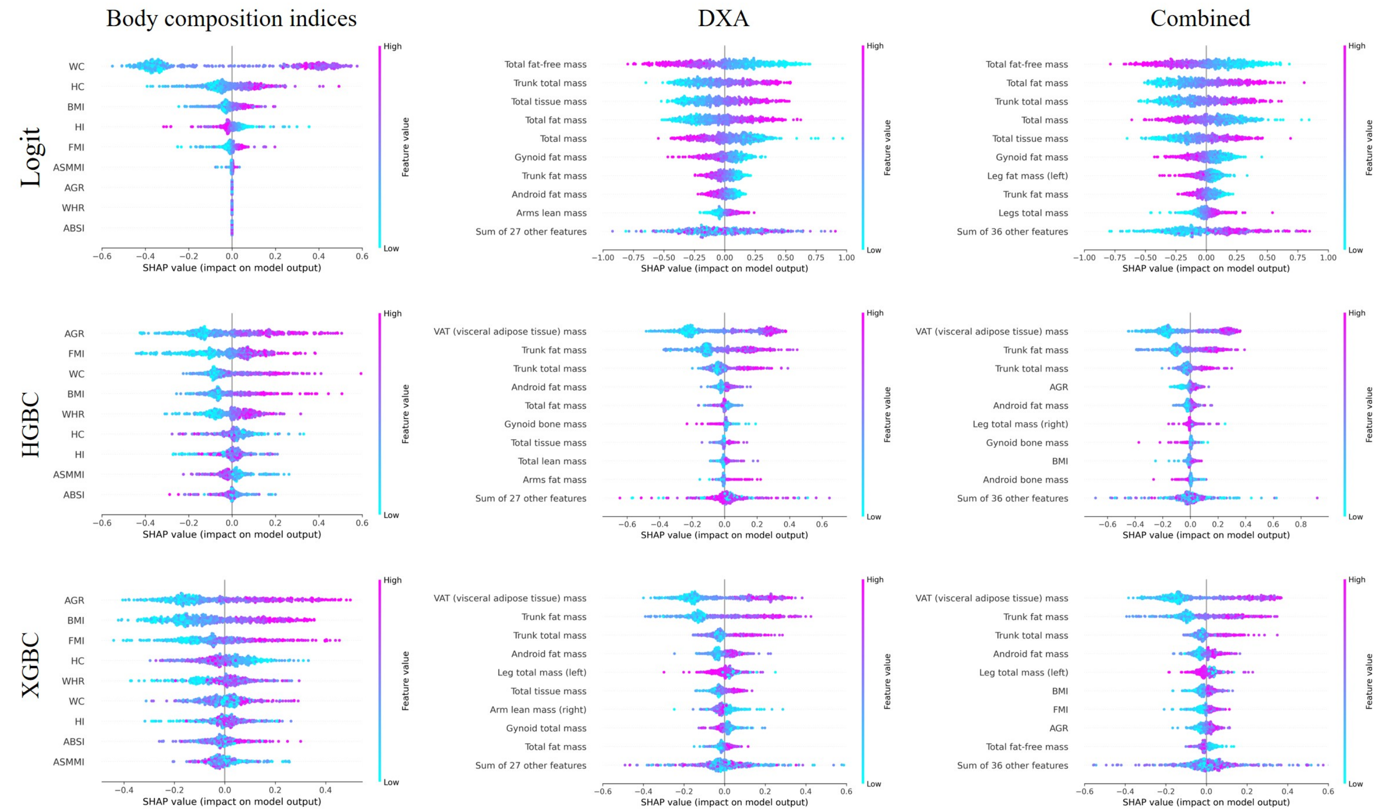

Supplementary Figure 5. SHAP feature importance of LR, HGBC, and XGBC models on binary classification. Evaluation is based on hold-out test sets.

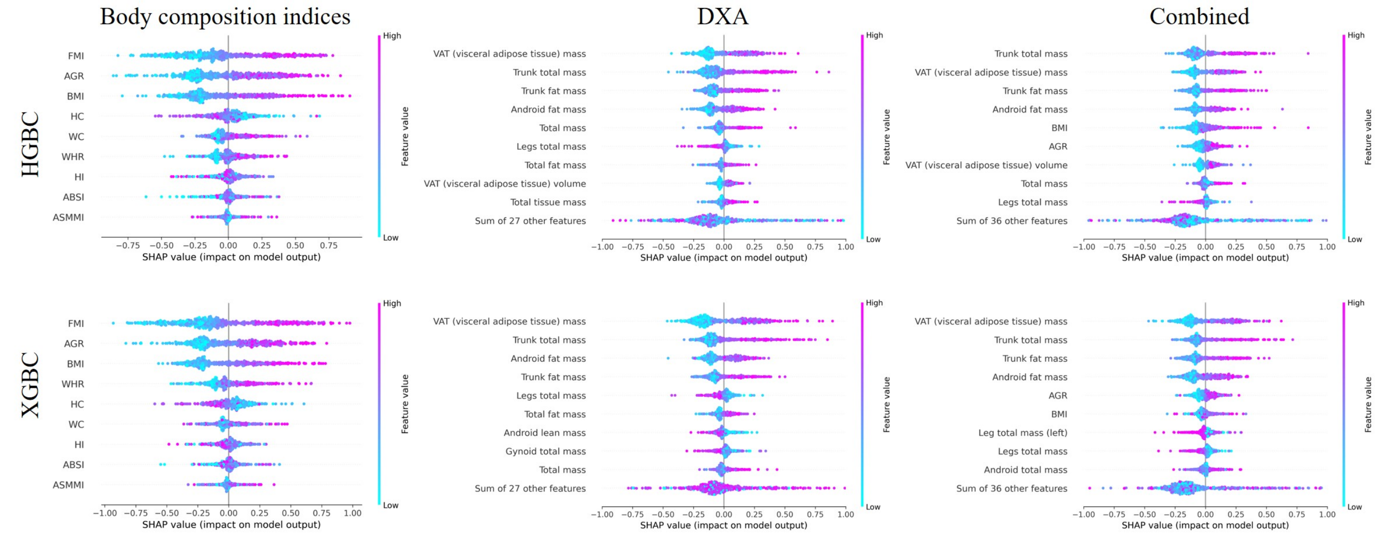

Supplementary Figure 6. SHAP feature importance of HGBC and XGBC on multiclass classification. Evaluation is based on hold-out test sets.

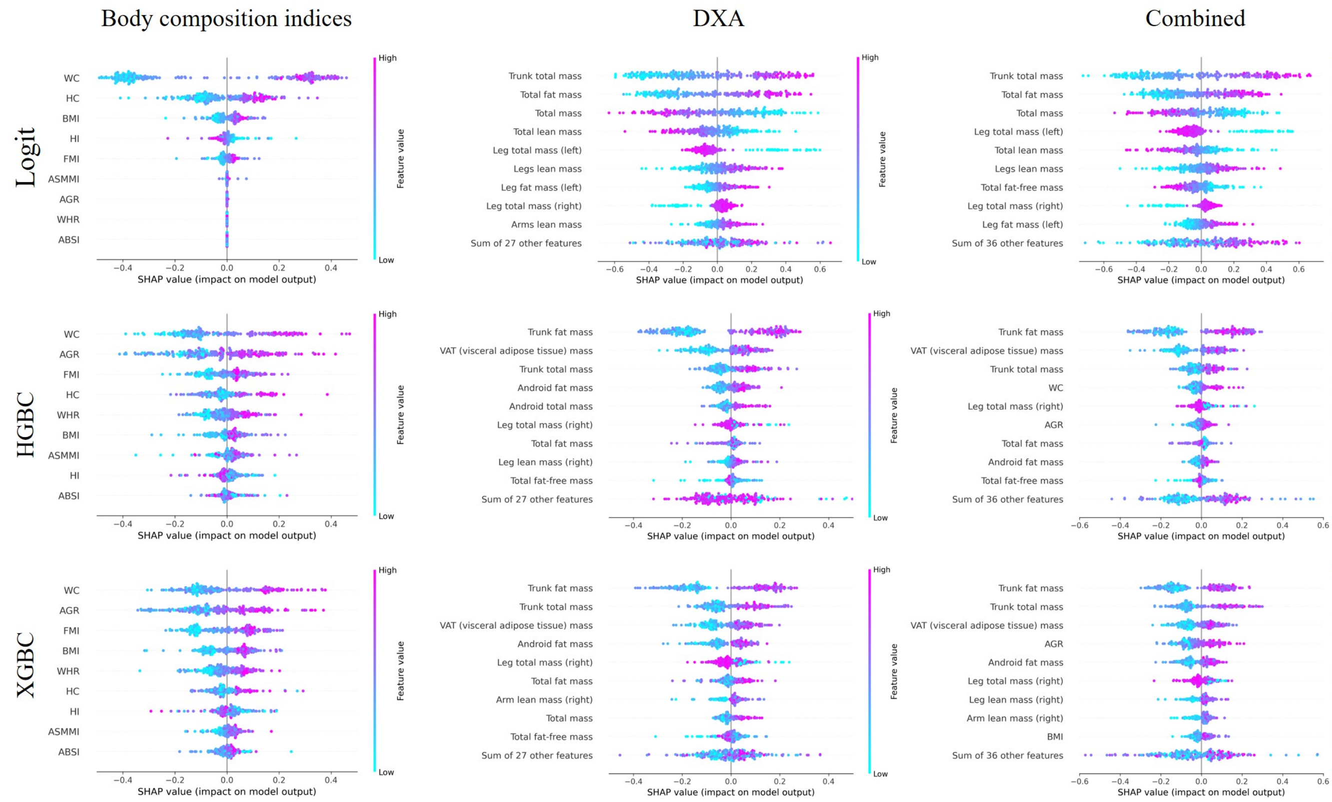

Supplementary Figure 7. SHAP feature importance of LR, HGBC and XGBC on binary classification for male. Evaluation is based on hold-out test sets.

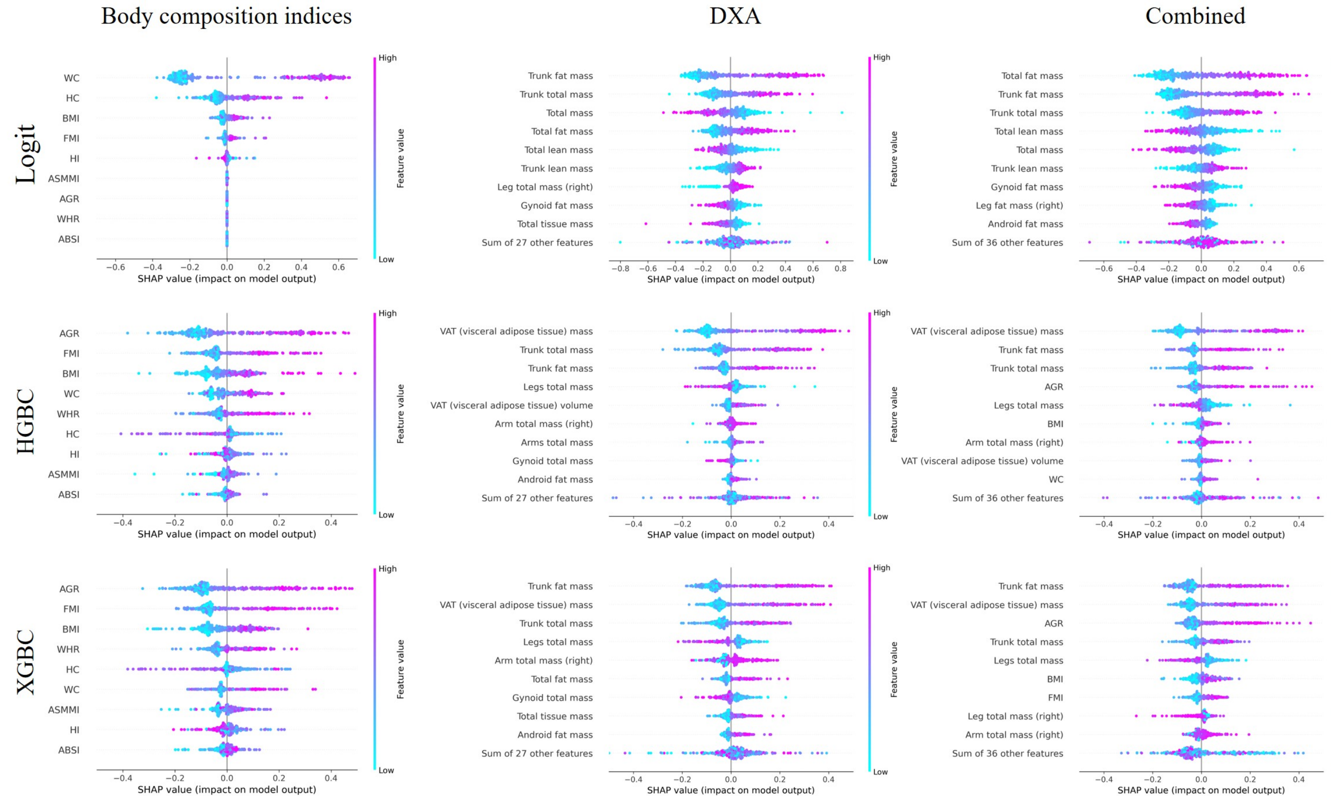

Supplementary Figure 8. SHAP feature importance of LR, HGBC and XGBC on binary classification for female. Evaluation is based on hold-out test sets.

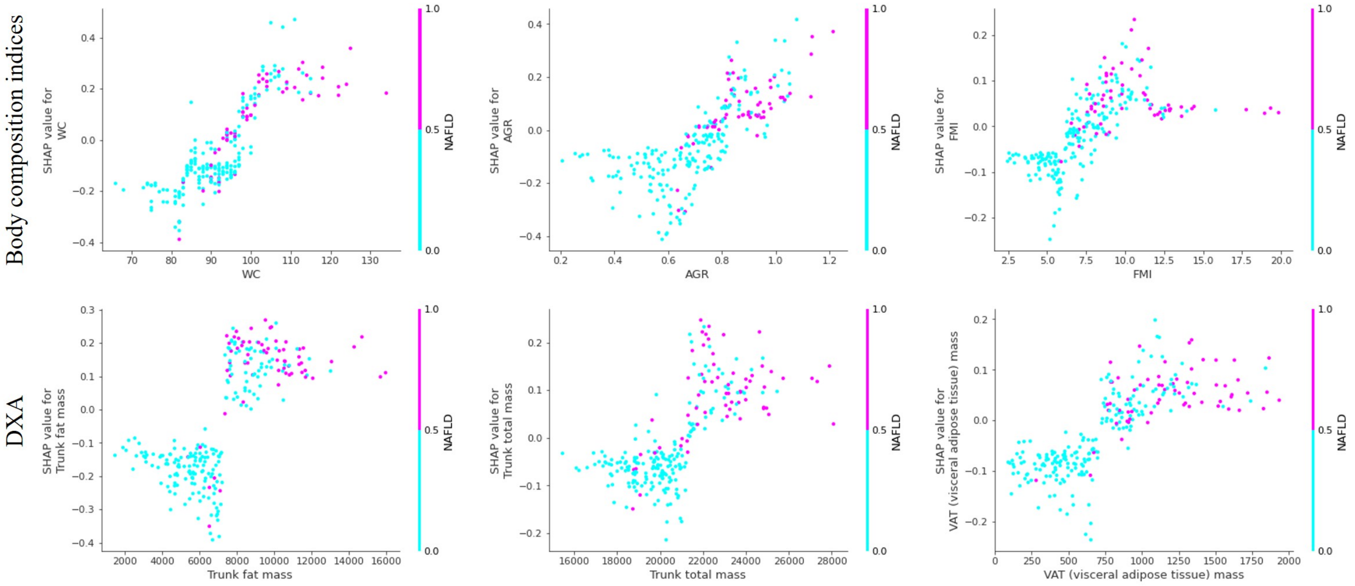

Supplementary Figure 9. SHAP dependence plots of the top 3 predictors of HGBC models for male.

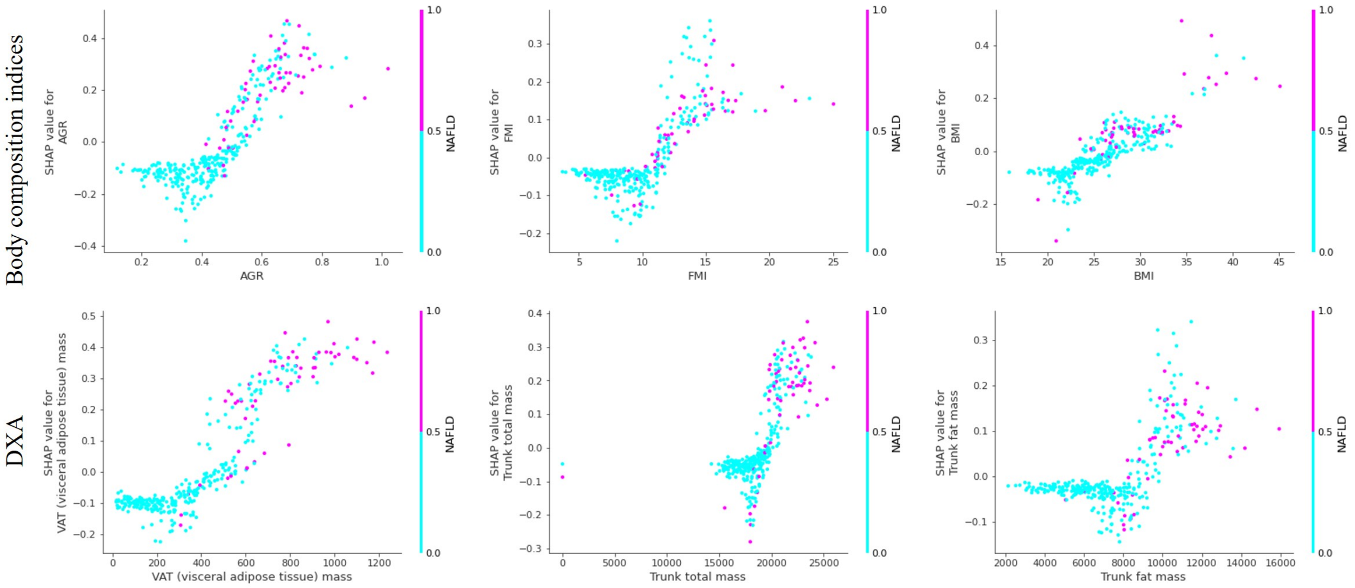

Supplementary Figure 10. SHAP dependence plots of the top 3 predictors of HGBC models for female.
